# Prevalence and diversity of avian haemosporidian parasites across islands of Milne Bay Province, Papua New Guinea

**DOI:** 10.1007/s00436-022-07490-y

**Published:** 2022-04-01

**Authors:** Wilmer Amaya-Mejia, Molly Dodge, Brett Morris, John P. Dumbacher, Ravinder N. M. Sehgal

**Affiliations:** 1 Department of Biology, San Francisco State University, 1600 Holloway Avenue, San Francisco, CA 94132 USA; 2 California Academy of Sciences, Golden Gate Park, San Francisco, CA 94118 USA

**Keywords:** Avian haemosporidians, Island biogeography, Papua New Guinea, Host specificity, Parasite biogeography

## Abstract

The taxonomically diverse and relatively understudied avifauna of Papua New Guinea’s (PNG) island archipelagos provide a unique ecological framework for studying haemosporidian parasite differentiation and geographic structure. We implemented molecular and phylogenetic analyses of partial mitochondrial DNA sequences to assess the host distribution of 3 genera of vector-transmitted avian blood parasites (*Plasmodium*, *Leucocytozoon* and *Haemoproteus*) across a range of islands off the southeastern tip of PNG. We identified 40 new lineages of haemosporidians, including five lineages belonging to *Leucocytozoon*, a genus not previously described in this region. *Leucocytozoon* infections were only observed on the larger, human-inhabited islands. Lineages belonging to *Haemoproteus* were diverse and had broad geographic distribution. Compared to the mainland, *Haemoproteus* parasites on the smaller, more distant islands had greater host specificity and lower infection prevalence. The black sunbird (*Leptocoma aspasia*), a commonly caught species, was shown to be a rare host for *Haemoproteus* spp. infections. Moreover, although birds of the genus *Pitohui* harbor a neurotoxin (homobatrachotoxin), they demonstrated an infection prevalence comparable to other bird species. The islands of PNG display heterogeneous patterns of haemosporidian diversity, distribution and host-specificity and serve as a valuable model system for studying host-parasite-vector interactions.

**Supplementary Information:**

The online version contains supplementary material available at 10.1007/s00436-022-07490-y.

## Introduction

Being relatively isolated, avian haemosporidian (Haemosporida, Haemoproteidae) parasite communities in oceanic archipelagos present a noteworthy model system to study ecological and evolutionary drivers of pathogen prevalence and diversity. While island biogeography correlates with avian populations (MacArthur and Wilson 1967; Andersen et al. 2014; Linck et al. 2016), previous studies into parasite systems on islands have observed conflicting support for biogeographic association (Fallon et al. 2003a, b, 2005; Ishtiaq et al. 2008; Olsson-Pons et al. 2015; Clark et al. 2016; Ellis et al. 2017). Although some island-parasite systems supported island biogeography theory (Fallon et al. 2005; Ishtiaq et al. 2008; Olsson-Pons et al. 2015; Clark et al. 2016; Ellis et al. 2017; Padilla et al. 2017), parasites on other island systems did not show a correlation with geography or environmental conditions. These parasite communities were, instead, significantly correlated with host community composition (Fallon et al. 2003a, b; Beadell et al. 2004; Santiago-Alarcon et al. 2008; Svensson-Coelho and Ricklefs 2011; Olsson-Pons et al. 2015; Clark et al. 2016; Clark et al. 2018; Humphries et al. 2019). To accurately understand the role of island biogeography on parasite communities, we must conduct additional island-specific studies that incorporate geography, environmental conditions and host communities.

In Papua New Guinea (PNG), the distribution and dispersal of haemosporidian parasites, potential endemic hosts and the role of insect vectors are largely understudied. The remote Louisiade archipelago of PNG offers a non-linear network of small islands which could hypothetically serve as stepping stones, allowing the dispersal of parasite populations to more distant islands (Ricklefs and Bermingham 2007). As a tropical region, the avian community composition of PNG is diverse. This diversity includes endemic hosts, such as the black sunbird (*Leptocoma aspasia*); the genus of poisonous birds, *Pitohui* (Mayr and Diamond 2001; Dumbacher et al. 1992); and many other species that are not well characterized, either phylogenetically or geographically (Filardi and Moyle 2005; Andersen et al. 2014; Linck et al. 2016). Diverse host communities can have equally diverse haemosporidian communities (Clark et al. 2014). This has been shown in the only published study exploring the genetic diversity of *Haemoproteus* and *Plasmodium* lineages in PNG (Beadell et al. 2004). The authors found *Haemoproteus* infections in 31% of birds and *Plasmodium* in 10%. *Haemoproteus* infections were not uniformly distributed across host families and exhibited strong host family-specificity. Since 2004, additional studies in the greater Australo-Papuan region have shown similar trends (Olsson-Pons et al. 2015; Clark et al. 2016; Goulding et al. 2016), but none has explored how the geographic structure of the Louisiade archipelago may influence this host-driven diversity. The application of our results could be limited by the diversity and number of endemic hosts of PNG; however, our results could serve to exemplify conclusions drawn from other island systems.

This investigation aimed to study biogeography and host-parasite assemblages of PNG. We tested for parasites belonging to the genera *Haemoproteus*, *Plasmodium* and *Leucocytozoon* in the avifauna occurring across 23 islands and the mainland of PNG. While *Haemoproteus* and *Plasmodium* parasites were previously detected on the mainland, ours was the first study to determine the presence of *Leucocytozoon* parasites in the PNG region. We use molecular and phylogenetic techniques to describe the host-parasite associations, the geographic distribution and host-specificity of parasite lineages identified from a subset of PNG island bird communities. Considering proximity and size of islands can affect the geographical structure of parasite assemblages by influencing avian movement and vectors (Fallon et al. 2005), we hypothesized that (1) island size, (2) island distance from the mainland and (3) the diversity of the avian hosts would correlate with haemosporidian prevalence and diversity. Additionally, we expected that lineages of *Haemoproteus* would be detected more readily in closely related host species than were *Plasmodium* lineages*.*

## Materials and methods

Fieldwork occurred from 30 January to 14 February 2009 in the lowlands near Mt. Bosavi (6°31′54.2″ S, 143°06′36.8″ E) on the mainland and on 23 islands of Milne Bay Province, PNG, from 8 October to 14 November 2009 and from 14 September to 2 November 2011. Approximately 50 μl of whole blood was drawn from each bird by brachial venipuncture and stored in lysis buffer for subsequent molecular analysis (Sehgal et al. 2001). One blood film was prepared from each bird. Slides were fixed in methanol in the field and stained with Giemsa in the laboratory. Low slide quality prevented use to determine morphospecies or parasitemia (Valkiūnas et al. 2008a, b).

### Parasite screening

DNA was extracted from whole blood using the Wizard® SV Genomic DNA Purification System (Madison, WI, USA). Extraction of amplifiable DNA was verified by polymerase chain reaction (PCR) targeting the avian brain-derived neurotrophic factor (Sehgal and Lovette 2003).

First, we detected *Haemoproteus* and *Plasmodium* spp. using two PCR protocols; both amplify the partial mitochondrial cytochrome *b* gene. The first protocol was a nested PCR (nPCR). The primers HaemNF and HaemNR2 were used in the first reaction, followed by HaemF and HaemR2 for the nested reaction (Waldenström et al. 2004). The second protocol used the primers set L15183 and H15730 described previously (Fallon et al. 2003a, b; Szymanski and Lovette 2005). The PCR products contain overlapping regions of the cty *b* gene, with the second set of primers targeting a downstream region. Using both protocols reduced sequencing errors in the overlapping region by requiring 100% identical sequence while increasing the cyt *b* gene sequence length up to 750 bp.

A nPCR determined *Leucocytozoon* spp. presence following the protocol described by Hellgren et al. (2004) with a modified annealing temperature set to 54.5 °C.

All PCR reactions were performed using *Accupower*® PCR PreMix (Oakland, CA, USA) in 20 or 25 μl reaction volumes. Samples were accompanied by negative (ddH_2_0) and positive controls (previously extracted samples that were tested and verified by microscopy) to detect contamination and confirm the success of the PCR. Products were run on a 1.8% agarose gel using 1× TBE and visualized with ethidium bromide under ultraviolet light.

PCR products were purified using ExoSap-IT following the manufacturer’s instructions (Cleveland, OH, USA). Both strands for the cyt *b* fragment were directly sequenced using BigDye® version 1.1 sequencing kit (Foster City, CA, USA) on an ABI Prism 3100™ automated sequencer (Foster City, CA, USA). The occurrence of double peaks on the chromatogram was used to identify mixed infections. Mixed infections that were not able to be distinguished were excluded from further analysis. Sequences were aligned using Sequencher 4.8 (Ann Arbor, MI, USA) and Geneious 7.1.9 (http://www.geneious.com). Our sequences were compared to published sequences available on GenBank using the BLAST algorithm. Aligned sequences were deemed unique when there was a difference of 1% or >4 bp (Bensch et al. 2009)

### Phylogenetics

Phylogenetic analysis was conducted independently on all three genera. Reference sequences included a combination of morphologically identified lineages and lineages previously observed in PNG. *Plasmodium relictum* was used as an outgroup in *Haemoproteus* and *Leucocytozoon* analyses, while *Haemoproteus columbae* served as outgroup for the *Plasmodium* analysis. High-quality *Haemoproteus* sequences consisting of 451-bp of a continuous fragment were analyzed. Analysis of sequences of *Plasmodium* had 382 bp, and *Leucocytozoon* had 291 bp.

Estimates of sequence divergence and algorithms were computed using Geneious 7.1.9. The software MrModelTest (Nylander et al. 2004) selected the models GTR + Γ for genus *Leucocytozoon* and GTR + Ι + Γ for the genus *Haemoproteus* and *Plasmodium*. Phylogenies of the partial cyt *b* sequences were generated in MrBayes version 3.2.6 (Ronquist and Huelsenbeck 2001) using 1 cold and 2 hot Monte Carlo Markov chains and sampled every 1000 generations over 3 million generations. In all, 25% of these were discarded as “burn-in,” and the remaining trees were used to construct a majority consensus tree and calculate posterior probabilities (*p.p.*) to determine individual clades.

### Statistical analyses

Prevalence was calculated as the number of individuals infected out of the total sample size. The 95% confidence intervals were calculated using the methods listed in Walther et al. (2015). In short, the qbeta function was run using base R version 1.2.5033 (RStudio Team 2019) with the shape 1 parameter set to the number of positive samples +1 and the shape 2 parameter set to total sample size — positive samples +1.

Generalized linear-mixed effect models (GLMM) were conducted to test for effects on prevalence across islands. Each model was designed with binomial errors. Two full models were run using all samples from host species and islands. The first model was used to observe the effects of island geography on prevalence and included the terms island area, island distance from the PNG mainland and their interactions. This model had estimated Shannon diversity index set as a random variable to account for the linear relationship between geography and avian diversity. Sample sizes were set as weighted values. The second model was conducted to observe the effect of host diversity on prevalence. In this second model, estimated Shannon diversity was set as a fixed variable. Area and distance from the mainland of each island were set as random variables; the sample size was used as a weighted value.

Community diversity was quantified using the estimated completeness curve and Hill numbers. Diversity metrics were (1) species richness, (2) Shannon Index and (3) Simpson Index values (Chao et al. 2014). Values were calculated and visualized for islands (*n* >1) using the “iNEXT” package (http://chao.stat.nthu.edu.tw/wordpress/software_download/) for *Haemoproteus*, *Plasmodium* and avian host populations.

Host-parasite specificity index (S_TD_*) for each lineage of *Haemoproteus* and *Plasmodium* was calculated using the program TAXOBIODIV2 (http://www.otago.ac.nz/parasitegroup/downloads.html). Assigned values ranged from 1 to 4, with lower S_TD_* indicating greater host-specificity. Lineages detected multiple times in a single host species were given a default value of 1, and lineages only detected once were excluded. Mean S_TD_* values were calculated for both genera of parasites to quantify their overall host-specificity (Poulin and Mouillot 2005) and compared between genera using a Welch two-sample *t*-test.

We compared host-specificity and prevalence of *Haemoproteus* lineages between islands and the mainland. *T*-tests were conducted for the S_TD_* values and the prevalence of lineages found on the mainland compared to smaller, more distant islands (Loiseau et al. 2017).

## Results

**Fig. 1:**
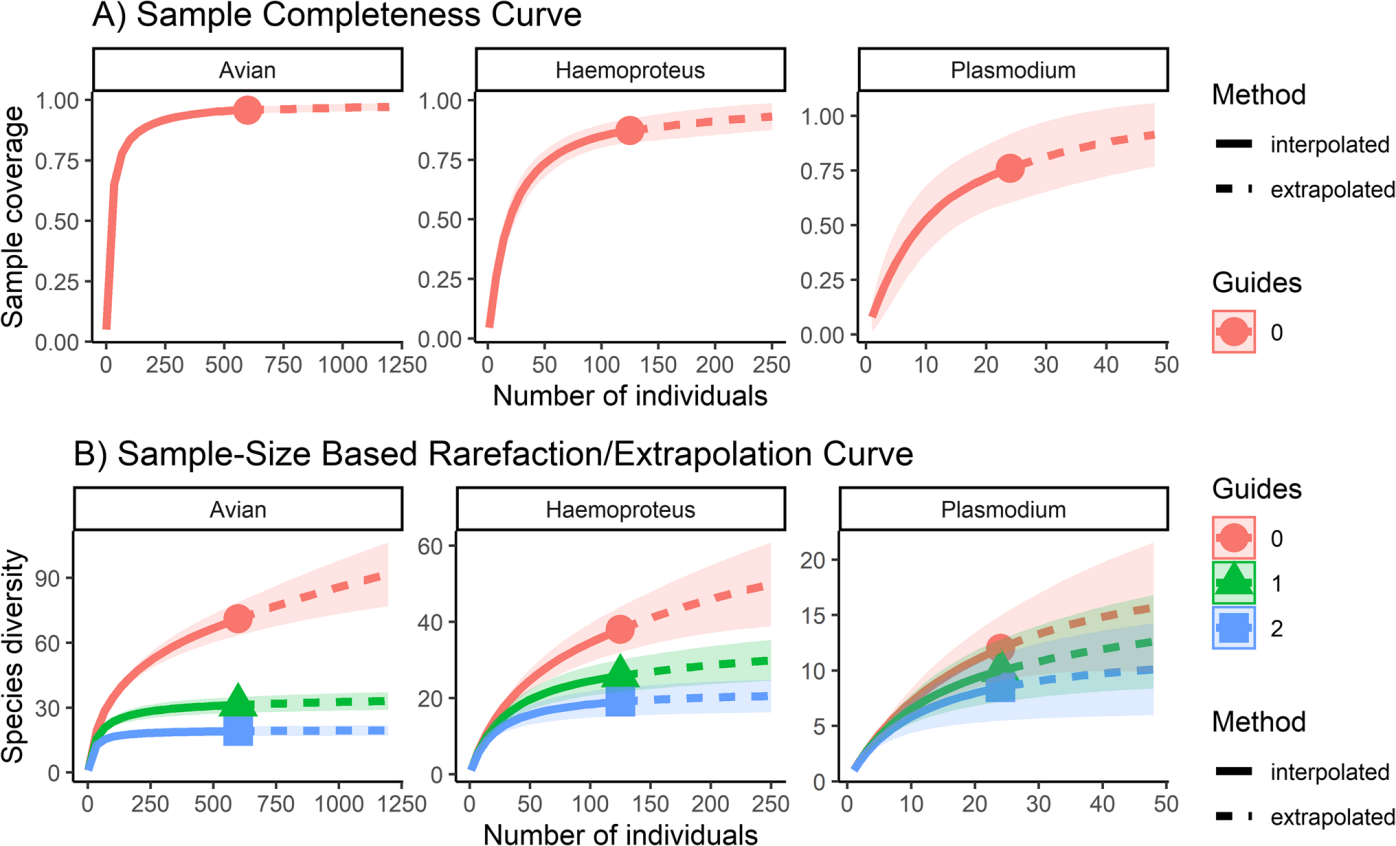
Species diversity estimates. (**A**) Sample completeness curve measured by sample coverage. (**B**) Sample size–based rarefaction and extrapolation curves for all avian species, Haemoproteus lineages and Plasmodium lineages collected across all sample sites in the Papua New Guinea region. Rarefaction curve is shown in solid line, sample size is shown as a solid dot and extrapolation curve is shown as a dashed line. Species richness (red), Shannon index (green) and Simpson index (blue) values are graphed

Using PCR and DNA sequencing, we screened 599 individual birds from 72 species (SI 1). A total of 126 individuals tested positive for parasites belonging to *Haemoproteus* (21%, C.I. 17.9–24.4%), 24 tested positive for parasites belonging to *Plasmodium* (4%, C.I. 2.7–5.9%) and 5 tested positive for parasites belonging to *Leucocytozoon* (0.8%, C.I. 0.5–1.9%). *Leucocytozoon* infections were found only on the large island of Bagaman (5.6%, C.I. 1.3–26%) and on the mainland (5.9%, C.I. 2.1–15.9%). No differences between infection prevalence at the family level were found (SI 2). Mixed infections were identified 36 times, 18 of which were not able to be isolated. A complete list of all relevant information for each lineage is provided in the Supplementary Information (SI 3). All novel sequences were deposited in GenBank (Accession JN792161-JN792180, MW271614-MW271619). Based on the sample completeness curve (Fig. 1A), we sampled >80% of the avian and *Haemoproteus* communities but <75% of the *Plasmodium* or *Leucocytozoon* parasite communities. Due to the small sample size of *Plasmodium* and *Leucocytozoon* infections, models were only created for the *Haemoproteus* communities.

### *Haemoproteus* prevalence and diversity

We identified 40 unique lineages of *Haemoproteus* in our study. The estimated *Haemoproteus* parasite richness was 63.395. The estimated Shannon and Simpson diversity indexes were 33.6 and 22.7, respectively (Fig. 1B).

**Fig. 2:**
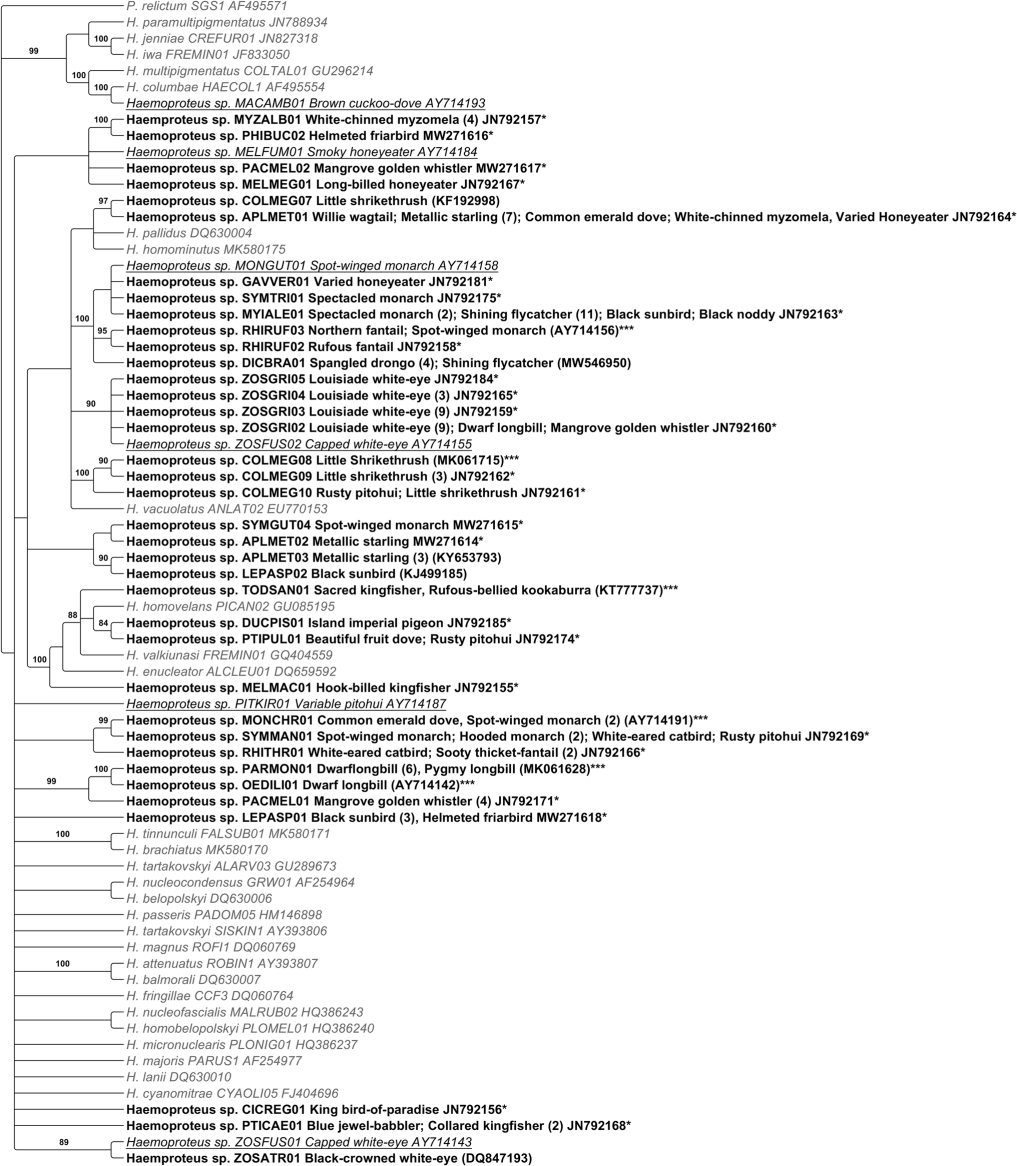
Phylogenetic tree of Papua New Guinea *Haemoproteus* parasite lineages based on 451 bp of the partial mitochondrial cytochrome *b* gene. Bayesian posterior probabilities >80% are shown. Lineage name, host species, number of infected hosts and GenBank accession number are shown for lineages observed in this study. General reference sequences are shown in grey, italicized text. Reference sequences obtained from Beadell et al. (2004) are shown in black, italicized and underlined. Sequences obtained from our study are in bolded, black text. Single asterisk indicates the sequences are considered novel with >1% difference from published sequences. Accession numbers inside of parentheses indicate the closest available sequence on GenBank (>99% identity). Triple asterisks indicate sequence matched with <1% difference to a published sequence obtained in the Australo-Papuan region

Lineages found in the Australo-Papuan region appear to be more closely related to each other than to lineages located around the world. Noticeable exceptions are the lineages TODSAN01, DUCPIS01, PTIPUL01 and MELMAC01, which showed similarities to the lineages PICAN02, FREMIN01 and ALCLEU01 (Fig. 2). We examined prevalence at species and family levels to account for the presence of Columbidae, which are rare hosts of the subgenus *Parahaemoproteus* (Martinsen et al. 2008*;* Križanauskienė et al. 2013). When comparing species (*n* >5), the prevalence of *Haemoproteus* spp. was highest in the dwarf longbill (*Oedistoma iliolophus*) (*n =* 10, 90%, C.I. 58.7–97.7%). In families (*n* >5), *Haemoproteus* spp. prevalence was highest in the family Melanocharitidae (*n =* 15, 66.7%, C.I. 41.3–84.8%). The little shrikethrush (*Colluricincla megarhyncha*) had the second-highest prevalence at the species and family level (*n = 13*, 46.2%, C.I. 23–71.1%).

### *Plasmodium* prevalence and diversity

**Fig. 3:**
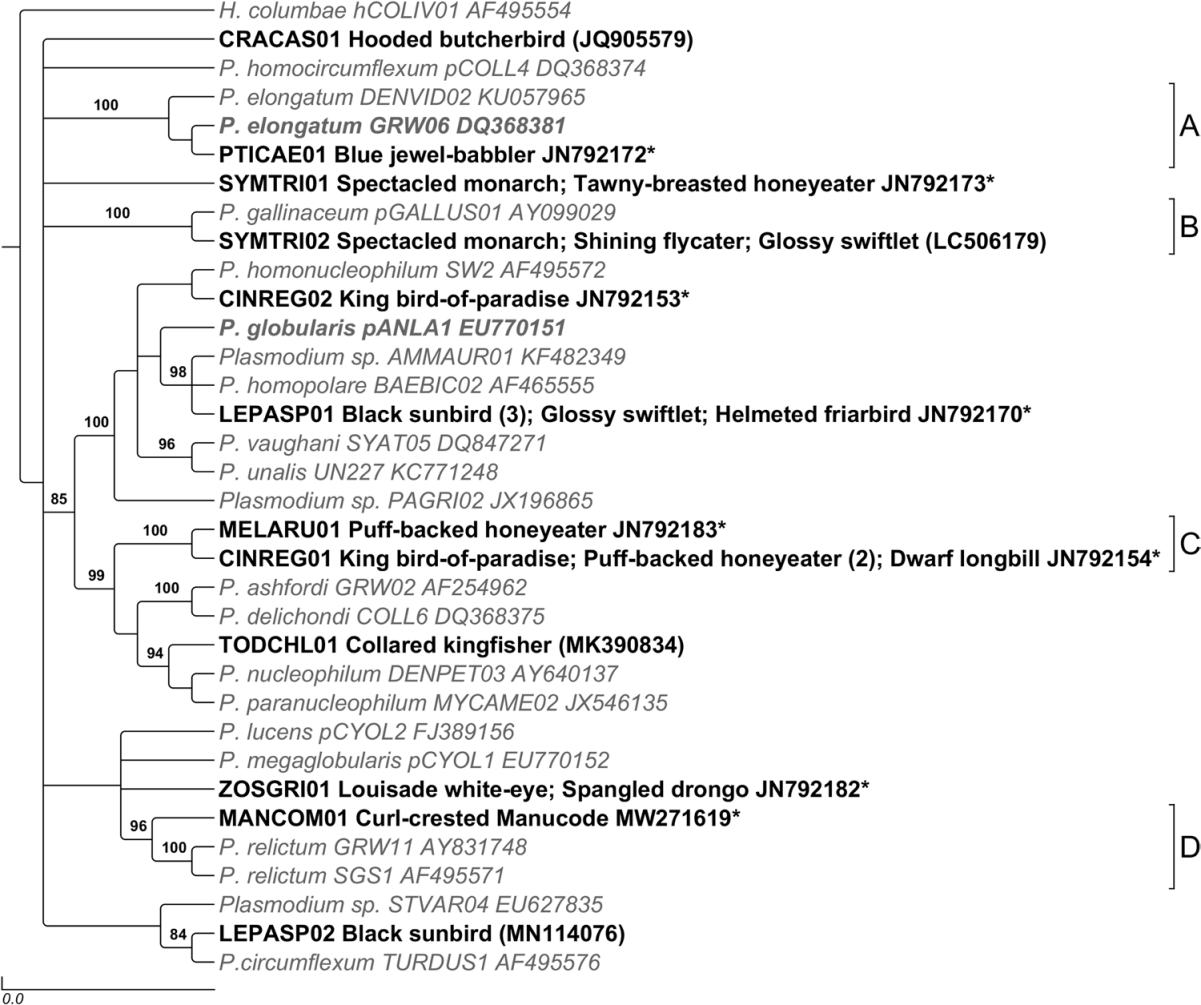
Phylogenetic tree of Papua New Guinea *Plasmodium* parasite lineages based on 382 bp of the partial mitochondrial cytochrome *b* gene. Bayesian posterior probabilities >80 are shown. Lineage name, host species, number of infected hosts and GenBank accession number are shown for lineages observed in this study. Reference sequences are shown in grey, italicized text. Sequences obtained from our study are in bolded, black text. Single asterisk indicates the sequences are considered novel with >1% difference from published sequences. Accession numbers inside of parentheses indicate the closest available sequence on GenBank (>99% identity). Triple asterisks indicate sequence matched with <1% difference to a published sequence obtained in the Australo-Papuan region

From the 24 positive *Plasmodium* spp. infections, we isolated sequences for 14 novel lineages, among which the raw pairwise sequence divergence ranged from 1.6 to 10.1% (Fig. 3). The helmeted friarbird (*Philemon buceroides*) had the highest prevalence (*n* = 6, 16%, C.I. 3.7–57.9%), followed by the dwarf longbill (*n* = 10, 10%, C.I. 2.3–41.3%). The families Columbidae (*n=* 50, 16%, C.I. 8.4–28.6%) and Meliphagidae (*n =* 42, 11.9%, C.I. 5.3–25.1%) had the highest prevalence. Total estimated *Plasmodium* species richness, Shannon diversity and Simpson diversity were lower than *Haemoproteus* at 17.7, 14.9 and 12.5, respectively (Fig. 1B).

### *Leucocytozoon* prevalence and diversity

*Leucocytozoon* spp. had the lowest overall prevalence (0.8%, 5/599 individuals) and were only detected once in each of five different species (SI 4). Four of these birds were captured on the mainland. One infected singing starling was captured on Bagaman island, 217 km from the mainland.

Sequence divergence among *Leucocytozoon* lineages ranged from 0.2 to 7.0%. Phylogenetic analysis showed that lineages were more closely related to each other than to any of the reference sequences (SI 4).

### Island biogeography and parasite communities

We examined the effects of island biogeography (i.e., distance from mainland and island area) and avian host communities on parasite communities. For *Haemoproteus* lineages, distance from the mainland was negatively correlated with the prevalence (*P* = 0.016) (Table 1), while estimated Shannon diversity was positively correlated (*P* < 0.001) (Table 2). Due to small sample sizes, we refrained from testing Plasmodium or Leucocytozoon parasite communities.

**Table 1:** Generalized linear mixed-effect model results of island geography, log of island area and distance from the mainland of the response variables *Haemoproteus* infection prevalence of all sampled birds from PNG. Variable significance was determined using chi-squared significance test

Variable	Estimate	Standard error	*z*-value	*P*-value
*Haemoproteus* infection prevalence				
Intercept	−1.012	0.243	−4.161	<0.001***
Dist. from mainland	−0.004	0.002	−2.407	0.016**
**Random effect:** estimated Shannon diversity				

*** indicate p-value <0.001, ** indicate p-value <0.05

**Table 2:** Generalized linear mixed-effect model results of host diversity, determined as estimated Shannon Diversity, of the response variables *Haemoproteus* infection prevalence of all sampled birds from PNG. Variable significance was determined using the chi-squared significance test

Variable	Estimate	Standard error	*z*-value	*P*-value
*Haemoproteus* infection prevalence				
Intercept	−1.781	0.149	−11.917	<0.001***
Estimated Shannon diversity	0.037	0.008	4.496	<0.001***
**Random effect:** log **(**island area)				
**Random effect:** dist. from mainland				

*** indicate p-value <0.001

*Haemoproteus* lineages showed a significantly lower mean S_TD_* (2.33 ± 1.07) compared to *Plasmodium* lineages (2.94 ± 0.75) based on Welch two-sample *t*-test (*t* = −3.0073, *df* = 29.54, *P* = 0.005) (SI 3).

**Fig. 4:**
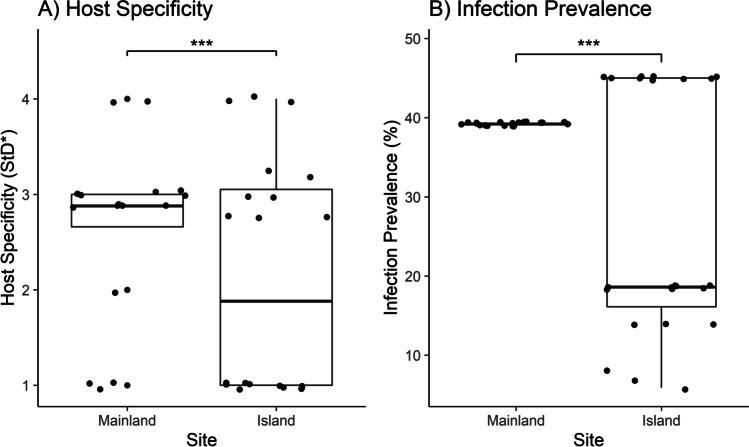
*T*-test of *Haemoproteus* spp. showing (**A**) host-specificity comparison between small, distant islands and the PNG mainland. Islands include Noapoi, Nuratu, Hummock, Nabwageta and Rara. Lower StD* indicates greater host specificity, and higher StD* indicates a generalist parasite. Islands showed greater host-specificity compared to the mainland. (**B**) Prevalence in small, distant islands and the PNG mainland. 95% confidence intervals of prevalence shown. *** indicate p-value <0.001

*Haemoproteus* lineages on smaller, distant islands (*n* = 18) had a lower S_TD_* (*t* = 3.65, *df* = 35, *P <* 0.001; Fig. 4A) and lower prevalence (*t* = −13.71, *df* = 42, *P <* 0.001) (Fig. 4B) compared to lineages found on the mainland (*n* = 15).

## Discussion

Our study focused on avian-parasite assemblages of the Louisiade Archipelago, PNG. We had lower haemosporidian prevalence (26%) compared to the previously observed prevalence (44%) (Beadell et al. 2004). Recent studies have determined that detection methods can bias *Haemoproteus* spp. over *Plasmodium* spp. during co-infections, an issue that may partially explain the relatively low detection of the latter (Ciloglu et al. 2019). This bias is an important consideration given the differences in protocols used during our study and the ones used by Beadell et al. (2004) (Neto et al. 2020). Infections spanned taxonomically divergent avifauna and several islands of PNG.

### Diversity and distribution of lineages

Our study revealed a noteworthy distribution of haemosporidians among avian hosts (SI 1). The southern variable pitohui (*Pitohui uropygialis*) and rusty pitohui (*Pseudocrectes ferrugineus*), endemic to PNG, were infected with *Leucocytozoon* (100%, C.I. 15.8–98.7%) and *Haemoproteus* (66.7%, C.I. 19.4–93.4%) parasites, respectively. These birds carry the potent neurotoxins batrachotoxins (BTX) on their skin and feathers (Dumbacher et al. 1992; Dumbacher et al. 2008). BTX can function as a chemical defense to repel or shorten the lifespan of chewing lice (order Phthiraptera) (Dumbacher 1999). Our results indicate that the toxin did not offer protection against haemosporidian vectors. Vectors can likely feed and transmit haemosporidian infections successfully before being affected by BTX. It is unclear whether the vectors are in contact for sufficient time to be affected by BTX or even susceptible to the toxin.

In contrast, black sunbirds, one of the most heavily sampled species, had relatively low prevalence (*n* = 56, *Haemoproteus* spp. 8.9%, C.I. 4–19.3%). Considering our current dataset, previous work that focused on sunbirds in Africa (Lauron et al. 2015), information relating to *Haemoproteus* parasites (Valkiūnas 2005) and trends on other island systems (Liao et al. 2016), it is unlikely that our results indicate transient infections or that infections are especially lethal to the black sunbirds. Instead, the ecological niche of black sunbirds, avoidance behaviours, microclimates, or a combination of these, offer alternative explanations. Future studies could explore these factors' contribution and prioritize high-quality blood smears to verify infections.

Previously, Columbiformes birds were thought to only be infected by parasites in the subgenus *Haemoproteus* (Martinsen et al. 2008). More recently, Columbiformes birds infected with *Parahaemoproteus* parasites have been identified (Križanauskienė et al. 2013; Schumm et al. 2021), and our results provide further evidence of these possible infections (Fig. 2).

One limitation to our study was the absence of data on vector populations. Vector distribution and feeding preferences regulate transmission and host-specificity of avian haemosporidians (Gager et al. 2008; Hellgren et al. 2008). Without this information, it is difficult to determine whether the absence of a particular genus of haemosporidians on each island was due to sample size or the absence of necessary vectors. For example, we did not find any infections belonging to the *Haemoproteus* subgenus, despite being previously observed in PNG (Beadell et al. 2004). This subgenus is transmitted by louse flies (family Hippoboscidae) and primarily infects members of the family Columbiformes (*n*= 50). We did detect a higher prevalence of *Parahaemoproteus* spp. infections on the mainland compared to the islands. This observation could indicate that the vectors, biting midges (family Ceratopogonidae), are more prevalent on the mainland than on the islands. Black flies (family Simuliidae), vectors of *Leucocytozoon* parasites, require high-quality running water for their early development (Lautenschläger and Kiel 2005). These conditions were absent from the smaller islands and could explain the low distribution of *Leucocytozoon* parasite infections. Future studies in this region could incorporate vector communities to provide insight into the feeding preferences of different vectors and to determine the potential consequences of novel vector introductions (Ricklefs et al. 2005; Tompkins and Gleeson 2006; Derraik et al. 2008; Howe et al. 2012; Ewen et al. 2012.).

### Relative phylogenetic relationships

Basal polytomy limits the interpretation of our phylogenetic tree. Despite this, our results do not conflict with recent publications focusing on haemosporidian phylogeny (Valkiūnas et al. 2020). *Haemoproteus* lineages in our study were closely related to each other and Australo-Papuan reference lineages, providing evidence that haemosporidian populations can be geographically isolated (Hellgren et al. 2015). In contrast, *Plasmodium* lineages were more heterogenous and demonstrated similarities with globally distributed lineages (Fig. 3).

The genus *Haemoproteus* is primarily comprised of host-specific lineages (Waldenström et al. 2002; Fallon et al. 2005), although there were initial observations of host-generalist lineages (Križanauskienė et al., 2006). Many *Haemoproteus* lineages within our study appeared more host-specific than *Plasmodium* lineages (*P* = 0.005), but both genera included generalist lineages. This supports recent observations of generalist *Haemoproteus* lineages in hosts (Nilsson et al. 2016) and vector populations of the southwest pacific islands (Ishtiaq et al. 2008). Eleven *Haemoproteus* lineages infected taxonomically distinct host families, and a few were also geographically distant (Fig. 2). Blood smears would be necessary to determine if detection of lineages in multiple species reflect established infections, unique crossover events or transient infections (Atkinson 1986).

### Island biogeography and parasite communities

Our data suggest that island biogeography was not solely responsible for shaping parasite communities of the Louisiade archipelago. Mainland PNG and Normanby Island, the largest island in the archipelago and closest to the mainland, had the highest *Haemoproteus* spp. prevalence. Forty percent of *Haemoproteus* lineages were endemic to one island. In contrast, only 27% of all *Plasmodium* lineages were endemic to a single island, with 18% of lineages shared between the mainland and the islands.

Our GLMM suggests that an island’s host community and the distance from the mainland individually affected *Haemoproteus* spp. prevalence (Tables 1 and 2). Similarly, Beadell et al. (2004) and Olsson-Pons et al. (2015) found that *Haemoproteus* prevalence was significantly related to host diversity. Loiseau et al. (2017) observed lineages on islands to be either generalist, shared with lineages on the mainland, or lineages with narrow niches. The lineages we observed on islands did not overlap with mainland lineages and instead were more often specialist (Fig. 4A). Whether this resulted from a small sample size or specialization over time requires high-quality slides and additional studies into the complete phylogenetic history of PNG lineages.

## Conclusions

Our study expands current knowledge of the haemosporidian communities found in Milne Bay Province, PNG, and explores the correlation between parasite community metrics, geographic characteristics and host communities. We provide the first account of *Leucocytozoon* parasite infections in PNG while providing additional data on *Haemoproteus* and *Plasmodium* lineages in the region. We observed that geography and host communities could contribute to the distribution of *Haemoproteus* parasites throughout PNG islands. These results could expand with future studies that address island geography, abiotic characteristics and physical conditions. The inclusion of these variables can explain how geography, hosts and even vector populations regulate haemosporidian distribution with implications relating to future habitat change.

## Supplementary information

ESM 1 (CSV 14 kb)

ESM 2 (CSV 6 kb)

ESM 3 (CSV 14 kb)

ESM 4 Phylogenetic tree of Papua New Guinea *Leucocytozoon* parasite lineages based on 291 bp of the partial mitochondrial cytochrome *b* gene. Bayesian posterior probabilities >80 are shown. Lineages listed in black correspond to those recorded in this study. Lineage name and GenBank accession numbers for all lineages are listed. Host species are only listed for lineages observed in this study. Lineages marked with * indicate sequences considered novel (>1% difference from published sequences). (PNG 236 kb)

High resolution image (TIFF 1651 kb)

## Data Availability

All data generated or analysed during this study are included in this published article and its supplementary information files.

## References

[CR1] Andersen MJ, Nyári ÁS, Mason I, Joseph L, Dumbacher JP, Filardi CE, Moyle RG (2014). Molecular systematics of the world’s most polytypic bird: the *Pachycephala pectoralis/melanura* (Aves: Pachycephalidae) species complex. Zool J Linnean Soc.

[CR2] Atkinson CT (1986). Host specificity and morphometric variation of *Haemoproteus meleagridis* Levine, 1961 (Protozoa: Haemosporina) in gallinaceous birds. Can J Zool.

[CR3] Beadell JS, Gering E, Austin J, Dumbacher JP, Peirce MA, Pratt TK, Atkinson CT, Fleischer RC (2004). Prevalence and differential host-specificity of two avian blood parasite genera in the Australo-Papuan region. Mol Ecol.

[CR4] Bensch S, Hellgren O, Perez-Tris J (2009). MalAvi: a public database of malaria parasites and related haemosporidians in avian hosts based on mitochondrial cytochrome b lineages. Mol Ecol Resour.

[CR5] Chao A, Gotelli NJ, Hsieh TC, Sande EL, Ma KH, Colwell RK, Ellison AM (2014). Rarefaction and extrapolation with Hill numbers: a framework for sampling and estimation in species diversity studies. Ecol Monogr.

[CR6] Ciloglu A, Ellis VA, Bernotienė R, Valkiūnas G, Bensch S (2019). A new one-step multiplex PCR assay for simultaneous detection and identification of avian haemosporidian parasites. Parasitol Res.

[CR7] Clark NJ, Clegg SM, Lima MR (2014). A review of global diversity in avian haemosporidians (*Plasmodium* and *Haemoproteus:* Haemosporida): new insights from molecular data. Int J Parasitol.

[CR8] Clark NJ, Wells K, Dimitrov D, Clegg SM (2016). Co-infections and environmental conditions drive the distribution of blood parasites in wild birds. J Anim Ecol.

[CR9] Clark NJ, Clegg SM, Sam K, Goulding W, Koane B, Wells K (2018). Climate, host phylogeny and the connectivity of host communities govern regional parasite assembly. Divers Distrib.

[CR10] Derraik JGB, Tompkins DM, Alley MR, Holder P, Atkinson T (2008). Epidemiology of an avian malaria outbreak in a native bird species (Mohoua ochrocephala) in New Zealand. J R Soc NZ.

[CR11] Dumbacher JP (1999). Evolution of toxicity in pitohuis: I. Effects of homobatrachotoxin on chewing lice (order Phthiraptera). Auk.

[CR12] Dumbacher JP, Beehler BM, Spande TF, Garroffo HM (1992). Homobatrachotoxin in the Genus Pitohui - chemical defense in birds. Science.

[CR13] Dumbacher JP, Deiner K, Thompson L, Fleischer RC (2008). Phylogeny of the avian genus Pitohui and the evolution of toxicity in birds. Mol Phylogenet Evol.

[CR14] Ellis VA, Medeiros MCI, Collins MD, Sari EHR, Coffey ED, Dickerson RC, Lugarini C, Startford JS, Henry DR, Merill L, Matthews AE, Hanson AA, Roberts JR, Joyce M, Kunkel MR, Ricklefs RE (2017). Prevalence of avian haemosporidian parasites is positively related to the abundance of host species at multiple sites within a region. Parasitol Res.

[CR15] Ewen JG, Bensch S, Blackburn TM, Bonneaud C, Brown R, Cassey P, Clarke RH, Pérez-tris J (2012). Establishment of exotic parasites: the origins and characteristics of an avian malaria community in an isolated island avifauna. Ecol Lett.

[CR16] Fallon SM, Bermingham E, Ricklefs RE (2003). Island and taxon effects in parasitism revisited: avian malaria in the Lesser Antilles. Evol.

[CR17] Fallon SM, Ricklefs RE, Swanson BL, Bermingham E (2003). Detecting avian malaria: an improved polymerase chain reaction diagnostic. J Parasitol.

[CR18] Fallon SM, Bermingham E, Ricklefs RE (2005). Host specialization and geographic localization of avian malaria parasites: a regional analysis in the Lesser Antilles. Am Nat.

[CR19] Filardi CE, Moyle RG (2005). Single origin of a pan-Pacific bird group and upstream colonization of Australasia. Nature.

[CR20] Gager AB, Del Rosario LJ, Dearborn DC, Bermingham E (2008). Do mosquitoes filter the access of Plasmodium cytochrome b lineages to an avian host?. Mol Ecol.

[CR21] Goulding W, Adlard RD, Clegg SM, Clark NJ (2016). Molecular and morphological description of *Haemoproteus* (*Parahaemoproteus*) *bukaka* (*species nova*), a haemosporidian associated with the strictly Australo-Papuan host subfamily Cracticinae. Parasitol Res.

[CR22] Hellgren O, Waldenstrom J, Bensch S (2004). A new PCR assay for simultaneous studies of *Leucocytozoon*, *Plasmodium*, and *Haemoproteus* from avian blood. J Parasitol.

[CR23] Hellgren O, Bensch S, Malmqvist B (2008). Bird hosts, blood parasites and their vectors; associations uncovered by molecular analyses of blackfly blood meals. Mol Ecol.

[CR24] Hellgren O, Atkinson CT, Bensch S, Albayrak T, Dimitrov D, Ewen JG, Soon Kim K, Lima MR, Palinauskas V, Ricklefs R, Sehgal RNM, Valkiūnas G, Tsuda Y, Marzal A (2015). Global phylogeography of the avian malaria pathogen *Plasmodium relictum* based on MSP1 allelic diversity. Ecography.

[CR25] Howe L, Castro IC, Schoener ER, Hunter S, Barraclough RK, Alley MR (2012). Malaria parasites (*Plasmodium* spp.) infecting introduced, native and endemic New Zealand birds. Parasitol Res.

[CR26] Humphries MB, Stacy MT, Ricklefs RE (2019). Population structure of avian malaria parasites. Ecol Evol.

[CR27] Ishtiaq F, Guillaumot L, Clegg SM, Phillimore AB, Black RA, Owens IP, Mundy NI, Sheldon BC (2008). Avian haematozoan parasites and their associations with mosquitoes across Southwest Pacific Islands. Mol Ecol.

[CR28] Križanauskienė A, Hellgren O, Kosarev V, Sokolov L, Bensch S, Valkiūnas G (2006). Variation in host specificity between species of avian hemosporidian parasites: evidence from parasite morphology and cytochrome B gene sequences. J Parasitol.

[CR29] Križanauskienė A, Iezhova TA, Sehgal RNM, Carlson JS, Palinauskas V, Bensch S, Valkiūnas G (2013). Molecular characterization of *Haemoproteus sacharovi* (Haemosporidia, Haemoproteidae), a common parasite of columbiform birds, with remarks on classification of haemoproteids of doves and pigeons. Zootaxa.

[CR30] Lauron EJ, Loiseau C, Bowie RCK, Spicer GS, Smith TB, Melo M, Sehgal RNM (2015). Coevolutionary patterns and diversification of avian malaria parasites in African sunbirds (Family Nectariniidae). Parasitol.

[CR31] Lautenschläger M, Kiel E (2005). Assessing morphological degradation in running water using Blackfly communities (Diptera, Simuliidae): can habitat quality be predicted from land use?. Limnologica.

[CR32] Liao W, Atkinson CT, LaPointe DA, Samuel MD (2016). Mitigating future avian malaria threats to Hawaiian forest birds from climate change. PLoS ONE.

[CR33] Linck E, Schaack S, Dumbacher JP (2016). Genetic differentiation within a widespread “supertramp” taxon: molecular phylogenetics of the Louisiade white-eye (*Zosterops grisotinctus*). Mol Phylogenet Evol.

[CR34] Loiseau C, Melo M, Lobato E, Beadell JS, Fleischer RC, Reis S, Doutrelant C, Covas R (2017). Insularity effects on the assemblage of the blood parasite community of the birds from the Gulf of Guinea. J Biogeogr.

[CR35] MacArthur RH, Wilson EO (1967). *The theory of island biogeography*.

[CR36] Martinsen ES, Perkins SL, Schall JJ (2008). A three-genome phylogeny of malaria parasites (Plasmodium and closely related genera): evolution of life-history traits and host switches. Mol Phylogenet Evol.

[CR37] Mayr E, Diamond J (2001). *The birds of Northern Melanesia: speciation*, *ecology*, *and biogeography*.

[CR38] Neto JM, Mellinger S, Halupka L, Marzal A, Zehtindjiev P, Westerdahl H (2020). Seasonal dynamics of haemosporidian (Apicomplexa, Haemosporida) parasites in house sparrows Passer domesticus at four European sites: comparison between lineages and the importance of screening methods. Int J Parasitol.

[CR39] Nilsson E, Taubert H, Hellgren O, Huang X, Palinauskas V, Markovets MY, Valkiūnas G, Bensch S (2016). Multiple cryptic species of sympatric generalists within the avian blood parasite *Haemoproteus majoris*. J Evol Biol.

[CR40] Nylander JAA, Ronquist F, Huelsenbeck JP, Nieves-Aldrey JL (2004). Bayesian phylogenetic analysis of combined data. Syst Biol.

[CR41] Olsson-Pons S, Clark NJ, Ishtiaq F, Clegg SM (2015). Differences in host species relationships and biogeographic influences produce contrasting patterns of prevalence, community composition and genetic structure in two genera of avian malaria parasites in southern Melanesia. J Anim Ecol.

[CR42] Padilla DP, Illera JC, Gonzalez-Quevedo C, Villalba M, Richardson DS (2017). Factors affecting the distribution of haemosporidian parasites within an oceanic island. Int J Parasitol.

[CR43] Poulin R, Mouillot D (2005). Combining phylogenetic and ecological information into a new index of host specificity. J Parasitol.

[CR44] Ricklefs RE, Bermingham E (2007). The causes of evolutionary radiations in archipelagos: Passerine birds in the Lesser Antilles. Am Nat.

[CR45] Ricklefs RE, Fallon SM, Latta SC, Swanson BL, Bermingham E, Greenberg R, Marra PP (2005). Migrants and their parasites: a bridge between two worlds. Birds of two worlds: the ecology and evolution of migration.

[CR46] Ronquist F, Huelsenbeck JP (2001). MRBAYES: Bayesian inference of phylogenetic trees. Bioinform.

[CR47] Rstudio Team (2019) RStudio: integrated development for R. *RStudio*, *Inc.*, Boston, MA. http://www.rstudio.com

[CR48] Santiago-Alarcon D, Whiteman NK, Parker PG, Ricklefs RE, Valkiūnas G (2008). Patterns of parasite abundance and distribution in island populations of Galapagos endemic birds. J Parasitol.

[CR49] Schumm YR, Bakaloudis D, Barboutis C, Cecere JG, Eraud C, Fischer D, Hering J, Hillerich K, Lormée H, Mader V, Masello JF, Metzger B, Rocha G, Spina F, Quillfeldt P (2021). Prevalence and genetic diversity of avian haemosporidian parasites in wild bird species of the order Columbiformes. Parasitol Res.

[CR50] Sehgal RNM, Lovette IJ (2003). Molecular evolution of three avian neurotrophin genes: implications for proregion functional constraints. J Mol Evol.

[CR51] Sehgal RNM, Jones HI, Smith TB (2001). Host specificity and incidence of *Trypanosoma* in some African rainforest birds: a molecular approach. Mol Ecol.

[CR52] Svensson-Coelho M, Ricklefs RE (2011). Host phylogeography and beta diversity in avian haemosporidian (Plasmodiidae) assemblages of the Lesser Antilles. J Anim Ecol.

[CR53] Szymanski MM, Lovette IJ (2005). High lineage diversity and host sharing of malarial parasites in a local avian assemblage. J Parasitol.

[CR54] Tompkins DM, Gleeson DM (2006). Relationship between avian malaria distribution and an exotic invasive mosquito in New Zealand. J R Soc NZ.

[CR55] Valkiūnas G (2005). Avian malaria parasites and other haemosporidia.

[CR56] Valkiūnas G, Iezhova TA, Križanauskienė A, Palinauskas V, Sehgal RN, Bensch S (2008). A comparative analysis of microscopy and PCR-based detection methods for blood parasites. J Parasitol.

[CR57] Valkiūnas G, Zehtindjiev P, Dimitrov D, Krizanauskiene A, Iezhova TA, Bensch S (2008). Polymerase chain reaction-based identification of *Plasmodium* (*Huffia*) *elongatum*, with remarks on species identity of haemosporidian lineages deposited in GenBank. Parasitol Res.

[CR58] Valkiūnas G, Ilgūnas M, Bukauskaitė D, Romeiro Fernandes Chagas C, Bernotienė R, Himmel T, Harl J, Weissenböck H, Iezhova TA (2020). Molecular characterization of six widespread avian haemoproteids, with description of three new *Haemoproteus* species. Acta Trop.

[CR59] Waldenström J, Bensch S, Kiboi S, Hasselquist D, Ottosson U (2002). Cross-species infection of blood parasites between resident and migratory songbirds in Africa. Mol Ecol.

[CR60] Waldenström J, Bensch S, Hasselquist D, Ostman O (2004). A new nested polymerase chain reaction method very efficient in detecting Plasmodium and Haemoproteus infections from avian blood. J Parasitol.

[CR61] Walther E, Carlson JS, Cornel A, Morris BK, Sehgal RNM (2015). First molecular study of prevalence and diversity of avian haemosporidia in a Central California songbird community. J Ornithol.

