# Supplementary figures and images for: Prevalence and diversity of avian haemosporidian parasites across islands of Milne Bay Province, Papua New Guinea
Source: Parasitol Res. 2022 Apr 1;121(6):1621–30. doi: 10.1007/s00436-022-07490-y (PMC9098550; doi:10.1007/s00436-022-07490-y)

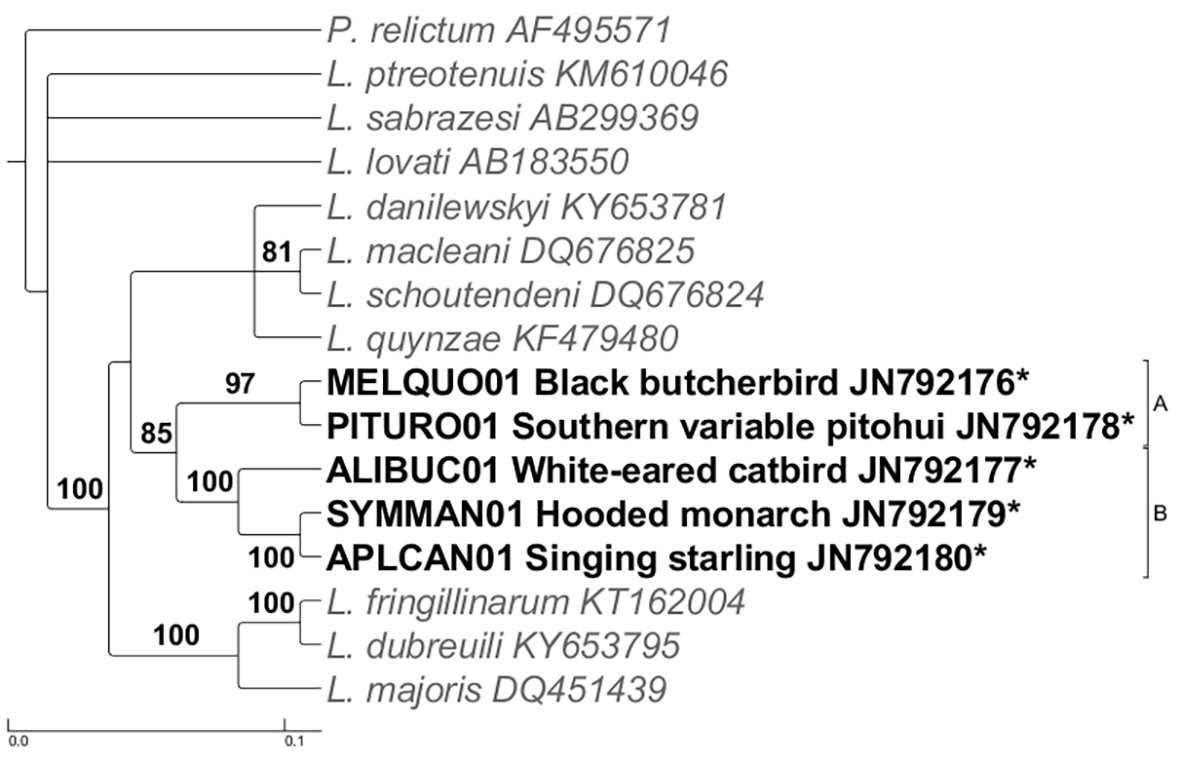

Supplement: Supplementary file 4 — Phylogenetic tree of Papua New Guinea Leucocytozoon parasite lineages based on 291 bp of the partial mitochondrial cytochrome b gene. Bayesian posterior probabilities >80 are shown. Lineages listed in black correspond to those recorded in this study. Lineage name and GenBank accession numbers for all lineages are listed. Host species are only listed for lineages observed in this study. Lineages marked with * indicate sequences considered novel (>1% difference from published sequences). (PNG 236 kb) [file 436_2022_7490_Fig5_ESM.png]

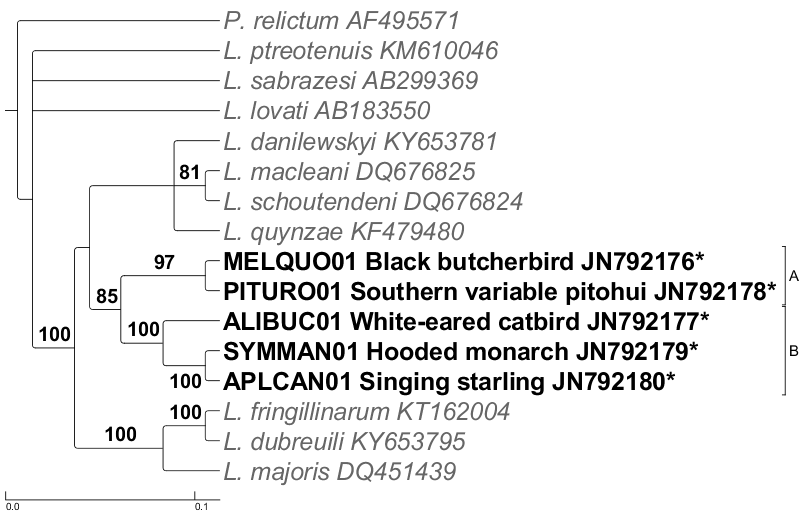

Supplement: Supplementary file 5 — High resolution image (TIFF 1651 kb) [file 436_2022_7490_MOESM4_ESM.tiff]
